# Supplementary material for: Multi‐dataset identification of innovative feature genes and molecular mechanisms in keratoconus
Source: J Cell Mol Med. 2024 Sep 19;28(18):e70079. doi: 10.1111/jcmm.70079 (PMC11412914; doi:10.1111/jcmm.70079)
Supplement: Supplementary file 2 — Material S2. [file JCMM-28-e70079-s002.zip › 7.GSEA analysis/5.RNF217-AS1/KEGG_PHENYLALANINE_METABOLISM.html]

Details for gene set KEGG\_PHENYLALANINE\_METABOLISM[GSEA]

|  || Dataset | RNF217-AS1.group.cls#h\_versus\_l |
| Phenotype | group.cls#h\_versus\_l |
| Upregulated in class | h |
| GeneSet | KEGG\_PHENYLALANINE\_METABOLISM |
| Enrichment Score (ES) | 0.5297195 |
| Normalized Enrichment Score (NES) | 1.4791028 |
| Nominal p-value | 0.02131783 |
| FDR q-value | 1.0 |
| FWER p-Value | 0.808 |
Table: GSEA Results Summary

  

Fig 1: Enrichment plot: KEGG\_PHENYLALANINE\_METABOLISM      
 Profile of the Running ES Score & Positions of GeneSet Members on the Rank Ordered List

  

| SYMBOL | TITLE | RANK IN GENE LIST | RANK METRIC SCORE | RUNNING ES | CORE ENRICHMENT || 1 | IL4I1 | na | 570 | 0.516 | 0.1904 | Yes |
| 2 | TAT | na | 1456 | 0.397 | 0.3242 | Yes |
| 3 | MAOB | na | 1741 | 0.370 | 0.4646 | Yes |
| 4 | PAH | na | 4025 | 0.251 | 0.4996 | Yes |
| 5 | AOC2 | na | 5673 | 0.193 | 0.5297 | Yes |
| 6 | DDC | na | 16555 | 0.003 | 0.2175 | No |
| 7 | GOT2 | na | 17814 | -0.009 | 0.1849 | No |
| 8 | MIF | na | 18730 | -0.018 | 0.1657 | No |
| 9 | MAOA | na | 19080 | -0.021 | 0.1639 | No |
| 10 | GOT1 | na | 20120 | -0.031 | 0.1465 | No |
| 11 | PRDX6 | na | 22072 | -0.052 | 0.1109 | No |
| 12 | ALDH3B2 | na | 23124 | -0.063 | 0.1060 | No |
| 13 | ALDH3A1 | na | 23827 | -0.073 | 0.1149 | No |
| 14 | ALDH3B1 | na | 24098 | -0.077 | 0.1379 | No |
| 15 | ALDH1A3 | na | 26655 | -0.119 | 0.1120 | No |
| 16 | HPD | na | 27994 | -0.149 | 0.1330 | No |
| 17 | AOC3 | na | 28131 | -0.152 | 0.1900 | No |
Table: GSEA details [plain text format]

  

Fig 2: KEGG\_PHENYLALANINE\_METABOLISM      
 Blue-Pink O' Gram in the Space of the Analyzed GeneSet

  

Fig 3: KEGG\_PHENYLALANINE\_METABOLISM: Random ES distribution      
 Gene set null distribution of ES for **KEGG\_PHENYLALANINE\_METABOLISM**
